# Composition Relation between Nonlinear Bloch Waves and Gap Solitons in Periodic Fractional Systems

**DOI:** 10.3390/ma11071134

**Published:** 2018-07-04

**Authors:** Liangwei Dong, Changming Huang

**Affiliations:** 1 Department of Physics, Shaanxi University of Science & Technology, Xi’an 710021, China; 2 Department of Electronic Information and Physics, Changzhi University, Changzhi 046011, China; hcm123_2004@126.com

**Keywords:** nonlinear optics, nonlinear Bloch waves, spatial optical solitons, defocusing Kerr medium, fractional Fourier transforms

## Abstract

Evolution of beams in nonlinear optical media with a fractional-order diffraction is currently attracting a growing interest. We address the existence of linear and nonlinear Bloch waves in fractional systems with a periodic potential. Under a defocusing nonlinearity, nonlinear Bloch waves at the centers or edges of the first Brillouin zone bifurcate from the corresponding linear Bloch modes at different band edges. They can be constructed by directly copying a fundamental gap soliton (in one lattice site) or alternatively copying it and its mirror image to infinite lattice channels. The localized truncated-Bloch-wave solitons bridging nonlinear Bloch waves and gap solitons are also revealed. We thus prove that fundamental gap solitons can be used as unit cells to build nonlinear Bloch waves or truncated-Bloch-wave solitons, even in fractional configurations. Our results provide helpful hints for understanding the dynamics of localized and delocalized nonlinear modes and the relation between them in periodic fractional systems with an optical nonlinearity.

## 1. Introduction

Periodic systems in optics always show a wide range of complex dynamics and intriguing properties [1,2]. In the linear case, according to the Bloch theorem, while infinite periodic waves are allowed in bands and prohibited in gaps, localized modes are forbidden anywhere. In the nonlinear case, however, spatially localized modes (solitons) can exist in the semi-infinite gap or gaps sandwiched between neighboring bands [3]. Besides solitons, periodic nonlinear Bloch waves (NLBWs) with infinite spatial scale and finite amplitudes are also supported by nonlinear systems with an optical lattice [4,5,6,7,8]. Such Bloch waves originate from the corresponding linear Bloch waves (LBWs) at the edges of bands in the first Brillouin zone (BZ).

Nonlinear Bloch waves are fundamentally important in the study of dynamics of nonlinear modes in various periodic physical systems. For example, self-trapped NLBWs were predicted in Bose-Einstein condensates (BECs) loaded into an external periodic potentials [4,5]. Truncated-Bloch-wave solitons associating with them were reported in [6]. In nonlinear optics, Zhang et al. revealed that the shrinkage of NLBWs results in the formation of multi-peaked solitons or fundamental solitons [7]. Nonlinear Bloch waves and their corresponding multi-peaked gap solitons were generalized into systems with a parity-time symmetry [8].

Typical nonlinear periodic systems include the nonlinear Shrödinger equation (NLSE) depicting gap solitons in optical lattices or waveguide arrays [1,2] and the Gross-Pitaevskii equation (GPE) describing BECs loaded into optical lattices [9,10]. The existence of localized nonlinear modes in gaps is due to the interplay between spatial periodicity and nonlinear dynamical evolution, even for a defocusing material nonlinearity or a repulsive interatomic interaction. Note that nonlinear Shrödinger equation can model the physics of wave dynamics in diverse fields, including deep water waves, rogue waves in water and optics, ion-acoustic waves in plasmas, BECs with an interatomic interaction, optical solitons in nonlinear media (fibres).

Recently, there is a growing interest in the physics of fractional Schrödinger equation (FSE), which was first put forward by N. Laskin in 2000 [11,12,13]. Fractional Schrödinger equation describes the physics when the Brownian trajectories in Feynman path integrals are replaced by Lévy flights. The interest in FSE is motivated, on the one hand by its fundamental importance for the dynamics of fractional-spin particles and the fractional field theory [11,12,13,14], and, on the other hand, by the rich possibilities they offer for simulating propagation dynamics of beams in optics [15].

In 2015, S. Longhi introduced FSE into optics and proposed an effective optical scheme to emulate the fractional quantum harmonic oscillator [15]. Following this work, there are a number of papers focusing on the propagation dynamics of beams in both linear [16,17,18,19] and nonlinear regimes [20,21,22]. In fractional dimensions, representative examples of the propagation dynamics of light beams include: chirped Gaussian beam propagation [16], PT symmetry [17], “accessible solitons” [18], propagation management [19], propagation of super-Gaussian beams [20], gap solitons [21], and solitons in PT symmetric potentials [22].

Until now, the properties of NLBWs in periodic fractional systems have not yet been explored. Recalling the fact that NLBWs in NLSE or GPE with an optical lattice can be regarded as infinite chains composed of fundamental gap solitons (FGSs) [7], one naturally asks: do NLBWs exist in a periodic system described by the nonlinear fractional Schrödinger equation (NLFSE)? If yes, another question arises: how they are influenced by the Lévy index of the system? Furthermore, are there any links between NLBWs and FGSs? Up to now, LBWs at the edges of the first and second bands of a fractional system were addressed only in [21]. The existence of nonlinear Bloch waves in fractional dimensions is still an open problem. The elucidation of NLBWs in NLFSE and their relation to FGSs is, therefore, a central goal of this paper.

## 2. Theoretical Model

We consider a one-dimensional nonlinear periodic fractional system described by the dimensionless equation in the normalized Cartesian coordinates *x* and ξ [21,23],

(1) $$i\frac{\partial \Psi }{\partial \xi }=\frac{1}{2}{\left(-\frac{\partial^{2}}{\partial x^{2}}\right)}^{\alpha /2}\Psi +{\left|\Psi \right|}^{2}\Psi +V\left(x\right)\Psi ,$$

where V(x) is an external periodic potential and ${\left(-\partial^{2}/\partial x^{2}\right)}^{\alpha /2}$ is the fractional Laplacian with α being the Lévy index (1<α≤2). In optics, the quantum Riesz derivative of order α stands for the fractional-order diffraction effect of a light beam. At α=2, Equation (1) degenerates to the conventional NLSE and a beam experiences a normal diffraction. If ξ is *z*, Equation (1) describes a beam propagating along the *z* direction in a defocusing medium [21,23]. When ξ is *t*, the equation describes the evolution of a condensate composed by fractional-spin particles loaded into optical lattices under a repulsive interparticle interaction [24]. For simplicity and comparison with [7], without loss of generality, we set ξ=z and V(x)=pcos(x) with *p* being the lattice depth.

The fractional Laplacian in Equation (1) can be realized experimentally by the scheme proposed by Zhang et al. [25]. Placing a well-designed phase mask between two convex lenses results in a phase change $\exp(-i/2|x{|}^{\alpha }z)$ at a certain propagation distance *z*. The first and second lenses play a role for transforming a beam into the inverse and real space. The optical lattice V(x) is usually obtained by interfering a pair of plane waves with appropriate input angles [1]. Inserting two identical crystals with a Kerr nonlinearity (e.g., KDP) beside the phase mask (very thin) and illuminating the setup with two interfering plane waves allow one to observe the beam propagation (under a fractional-order diffraction effect) in a Kerr medium modulated by an optical lattice.

For a typical input beam with wavelength λ=1μm, width $w_{0}=10\;\mu$m, by assuming the refractive index $n_{0}=3$, x=1 and z=1 in the dimensionless Equation (1) correspond to ∼10μm in the transverse direction and ∼0.63 mm in the propagation direction. Lattice depth p=1 corresponds to a maximum variation of refractive index ∼$8\times 10^{-5}$.

Stationary solutions of Equation (1) can be solved by assuming Ψ(x,z) in the form of Ψ(x,z)=ψ(x)exp(−ibz), where the field profile ψ(x) and chemical potential or propagation constant *b* obey the following differential equation:

(2) $$\frac{1}{2}(-\frac{\partial^{2}}{\partial x^{2}}{)}^{\alpha /2}\psi +{\left|\psi \right|}^{2}\psi +V\psi =b\psi .$$

Profiles of nonlinear modes can be solved numerically by a squared-operator iteration method [26].

## 3. Band Gap Structures and Bloch Modes in Linear Fractional Systems

Before we discuss the characteristics of guided nonlinear modes, it is important to investigate the Floquet-Bloch spectrum and the LBWs of the corresponding linear system. According to the Bloch theorem, we assume $\psi \left(x\right)=\phi_{n,k}\left(x\right)\exp\left(ikx\right)$ with *n* and *k* being the band index and wave vector, respectively. The periodicity of $\phi_{n,k}\left(x\right)=\phi_{n,k}\left(x+T\right)$ allows one to consider the dispersion relation only in the first Brillouin zone −π/T≤k≤π/T. Expanding ϕ(x) and V(x) into series of plane waves: $\phi_{n,k}\left(x\right)=\sum_{q}C_{q}\exp\left(iK_{q}x\right)$ and $V\left(x\right)=\sum_{m}P_{m}\exp\left(iK_{m}x\right)$ and inserting them into Equation (2), after neglecting the nonlinear item, we obtain the following eigenvalue function:

(3) $$-\frac{1}{2}{\left|k+K_{q}\right|}^{\alpha }C_{q}+\sum_{m}P_{m}C_{q-m}=bC_{q}.$$

Here $K_{q}=2\pi q/T$ and $P_{m}=\frac{1}{T}\int_{0}^{T}V\left(x\right)\exp\left(-iK_{m}x\right)dx$. Based on Equation (3), the dispersion relation or band-gap spectrum of the fractional system can be solved numerically by the Fourier collocation method, see e.g., p. 390 [26].

**Figure 1:**
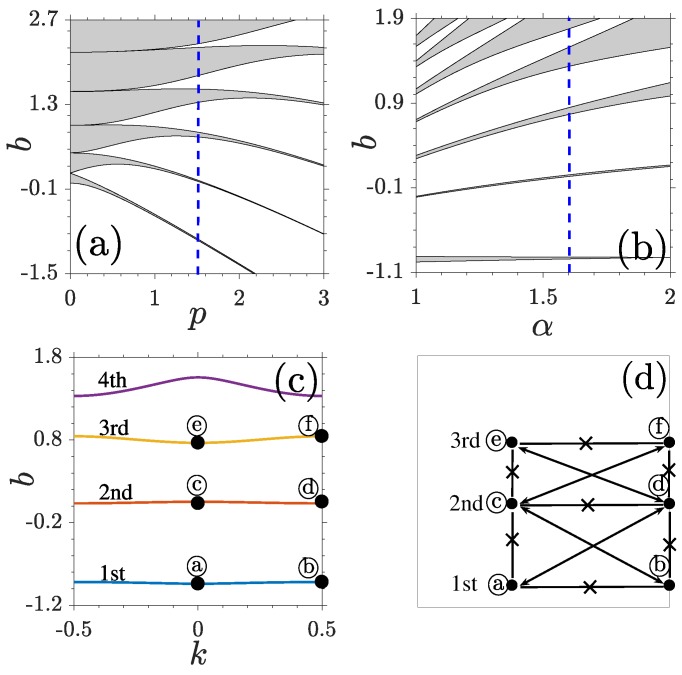
Bandgap structure at (**a**) α=1.6 and (**b**) p=1.5. (**c**) Bandgap spectrum at p=1.5 and α=1.6. (**d**) A simplified scheme for illustrating the symmetry relation between LBWs.

The band structures of a linear periodic system are displayed in Figure 1. At a fixed Lévy index, e.g., α=1.6, all finite gaps are closed if the lattice depth p=0, due to the absence of periodicity. With the increase of *p*, the 1st, 2nd, 3rd... gaps open up one by one (Figure 1a). For a fixed lattice depth, the variation of Lévy index also changes the band-gap structure of the system. While the first and second gaps shrink with the decrease of α, the higher gaps expand (Figure 1b). At the same time, finite bandgaps shift toward the direction of negative infinity.

Figure 1c shows an example of detailed band-gap spectra at p=1.5 and α=1.6. As *k* varies across the BZ, the corresponding $b_{n}\left(k\right)$ forms the bands. Infinite extended periodic LBWs are admitted in bands and the gaps sandwiched in the neighboring bands do not allow any physical solutions.

**Figure 2:**
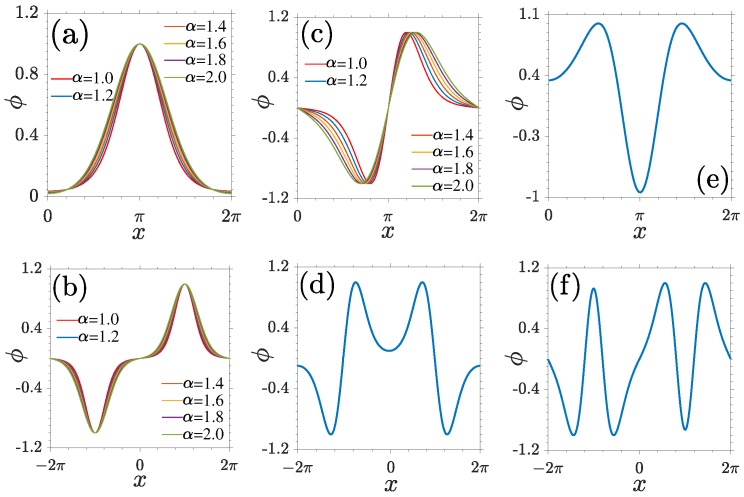
Normalized linear Bloch modes marked inFigure 1c at the centers (**a**,**c**,**e**) and the edges (**b**,**d**,**f**) of the BZ. α=1.6 in (**d**–**f**).

The Bloch modes at the centers and edges of the BZ are particularly important, due to their simple symmetries and the possibility that localized nonlinear modes can bifurcate from them. Typical examples of LBWs in one or two lattice periodicities at the lower and upper edges of the first, second, and third bands are plotted in Figure 2. We make four comments here. First, the LBWs at the centers of the BZ always occupy one lattice periodicity (Figure 2a,c,e) and LBWs at the edges fill two lattice channels (Figure 2b,d,f). Second, in the same band, the parity of the LBWs at the center is always opposite to that of the modes at the edge. The symmetries of center modes or edge modes between the neighboring bands are also opposite. The symmetry relation between LBWs can be understood by a simplified scheme shown in Figure 1d, where the lines with double arrows indicate a same symmetry and the lines overlapped with crosses manifest an opposite symmetry. Third, at the boundaries of one lattice periodicity, while the amplitudes of odd-symmetric LBWs descend to zero, the amplitudes of even-symmetric LBWs do not varnish. Fourth, LBWs shrink slowly and become more localized with the decrease of Lévy index α. This property is crucial for the stability of localized nonlinear modes originating from them [21].

## 4. Nonlinear Bloch Modes, Fundamental Gap Solitons, and Truncated-Bloch-Wave Solitons

When a defocusing nonlinearity is introduced, there are two types of stationary solutions. One is the localized gap solitons residing in the finite gaps and another is the nonlinear periodic Bloch waves bifurcating from the linear ones. Such nonlinear waves are extensive modes and spread towards infinity. For NLBWs, the strength of nonlinearity can be characterized by defining the norm in one lattice periodicity $N=\int_{0}^{2\pi }{\left|\phi_{k}\left(x\right)\right|}^{2}dx$. As to gap solitons, one usually uses the norm (also called power or energy flow) $N=\int_{-\infty }^{\infty }{\left|\phi \left(x\right)\right|}^{2}dx$ to feature the degree of nonlinearity.

**Figure 3:**
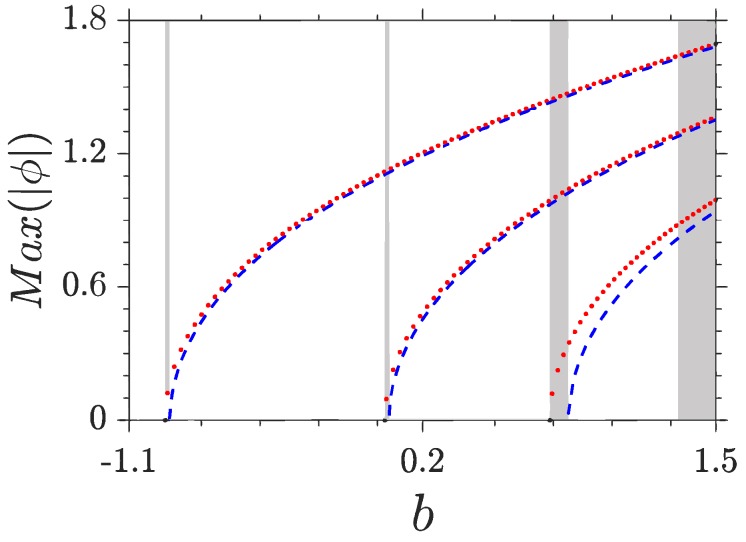
Amplitudes of NLBWs versus *b*. Shaded areas are linear bands and white regions are bandgaps. Dotted (red) and dashed (blue) lines denote NLBWs bifurcating from LBWs at the BZ centers and edges, respectively. p=1.5 and α=1.6.

Nonlinear Bloch waves can originate from all linear modes. In Figure 3, we show the amplitudes of nonlinear waves bifurcating from the centers and edges of the BZ for the first three bands. Note that the amplitudes of LBWs can be scaled to arbitrary values in the linear case. In contrast, NLBWs have finite amplitudes which cannot be scaled due to the presence of a nonlinearity. At centers or edges of the BZ, Equation (2) admits LBWs with infinitesimal amplitudes. As *b* increases, the amplitudes of NLBWs [max(|ϕ|)] grow monotonously and the LBW becomes a nonlinear one. We should point out that all branches of NLBWs can penetrate through bands freely, which is very different from localized gap solitons. With the growth of band index *n*, the difference between the amplitudes of NLBWs bifurcating from the lower and upper edges of bands becomes larger.

**Figure 4:**
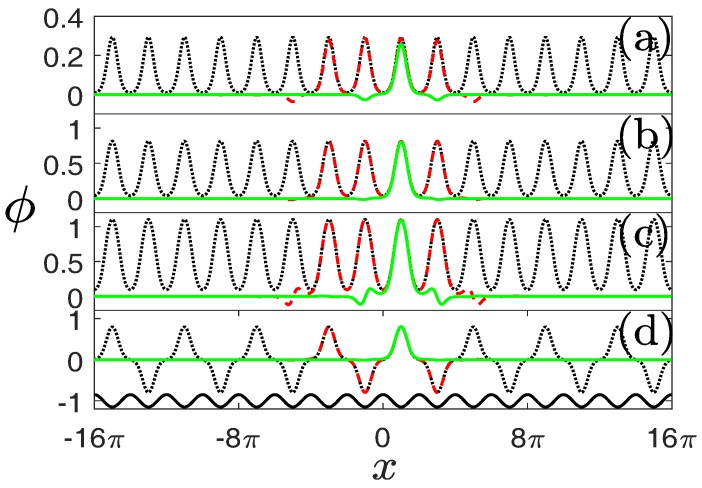
Nonlinear Bloch waves originating from the center (**a**–**c**) and edge (**d**) of the BZ and FGSs in the first band gap. Dotted (black), solid (green), and dashed (red) lines stand for NLBWs, FGS, and truncated-Bloch-wave solitons, respectively. (**a**) N=0.1317, b=−0.88; (**b**) N=1.2418, b=−0.45; (**c**) N=2.6653, b=0; (**d**) N=1.1506, b=−0.45. The solid curve at the bottom of (**d**) denotes the distribution of a lattice. α=1.6 and p=1.5 in all the panels.

Figure 4 displays the NLBWs in the first gap of the system with α=1.6. Compare the dotted curves in Figure 4a–c and LBWs shown in Figure 2a, one immediately finds that the distributions of NLBWs are the same as those of LBWs except for different amplitudes and widths. The nonlinear periodic system described by Equation (1) also admits gap solitons, among which the simplest case is FGSs. We observe from Figure 4a–c that the main lobes of FGSs coincide with the NLBWs in one lattice channel excellently. This implies that the NLBWs at the center of BZ can be constructed by piecing an infinite number of FGSs on a chain periodically. We thus preliminarily prove that the composition relation between NLBWs and FGSs in conventional GPE or NLSE [7] can be generalized into a fractional system.

This composition relation can be understood more clearly if we consider another type of localized gap solutions termed as “truncated-Bloch-wave solitons” [5,6,7,8]. While the main lobes of such nonlinear states match very well with the individual lobes of NLBWs and the main lobes of FGSs inside one lattice channel, their profiles beside the main lobes are completely identical with the profiles of FGSs in the region except for central lattice channel (Figure 4a–c). If one increases the number of peaks of such “big solitons” to infinity, NLBWs will be obtained. On the other hand, compressing big solitons into one lattice channel results in the formation of FGSs. Truncated-Bloch-wave soliton is, thus, an intermediate state bridging NLBWs and FGSs.

The construction of NLBWs and truncated-Bloch-wave solitons by FGSs also holds for nonlinear states originating from the edges of the BZ (Figure 4d). The only difference is that the FGS is pieced together with alternative signs. Recalling the LBWs shown in Figure 2b, the periodicity of NLBWs is two times that of the periodicity of NLBWs at the centers of the BZ. We should note that the peaks of NLBWs always sit on the maxima of external potentials.

**Figure 5:**
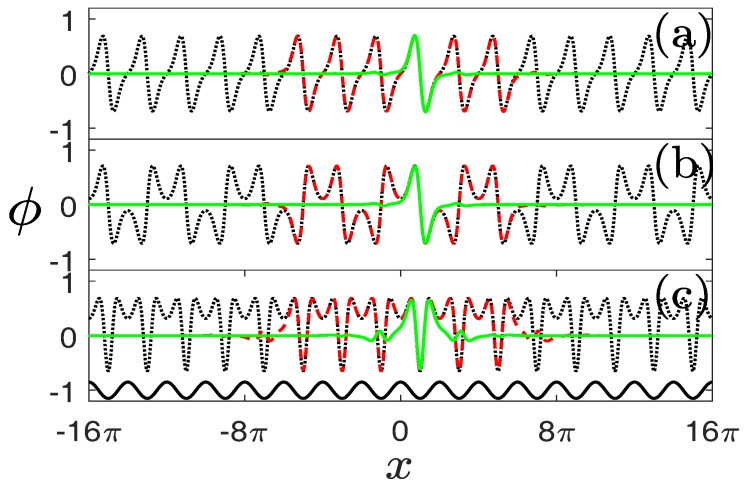
Nonlinear Bloch waves originating from the centers (**a**,**c**) and edge (**b**) of the BZ and FGSs in the second (**a**,**b**) and third (**c**) gaps. Dotted (black), solid (green), and dashed (red) lines stand for NLBWs, FGS, and truncated-Bloch-wave solitons, respectively. (**a**) N=1.1066; (**b**) N=1.2411; (**c**) N=1.5319; b=0.4 in (**a**,**b**) and 1.1 in (**c**). The solid curve at the bottom of (**c**) denotes the distribution of a lattice. α=1.6 and p=1.5 in all the panels.

Next, we focus on the NLBWs in the second bandgap. In this case, the FGSs (also termed as subfundamental gap solitons in [4]) are different from those in the first gap. It contains two main out-of-phase lobes in one lattice channel and its profile inside the lattice channel cross one time of the *x* axis (Figure 5a,b). At the center of the BZ, repeating a FGS over the whole lattice will lead to the formation of an odd-symmetric NLBW (Figure 5a). In contrast, at the edge of the BZ, copying the FGS and its mirror image alternatively into all lattice channels results in an even-symmetric NLBW (Figure 5b). The periodicity at the edge of the BZ is again two times of that at the center of the BZ. With the decrease of Lévy index α, the slope between two main lobes of a FGS becomes steep due to the contraction of LBWs (see e.g., Figure 2c).

Even- and odd-symmetric NLBWs can be constructed by the repetitions of a FGS in the third gap at the center and edge of the BZ. Now, the FGS exhibits a new form. It is even-symmetric and crosses the *x* axis two times in one lattice channel (Figure 5c). We therefore draw a conclusion: the profiles of FGWs used as fundamental units in the *n*th gap have n−1 nodes in one lattice cell. Directly copying it or alternatively copying it and its mirror image over the whole lattice will obtain the NLBWs at the center and edge of the BZ. One can see clearly the excellent matching between the NLBWs, FGSs, and truncated-Bloch-wave solitons, even in higher gaps.

**Figure 6:**
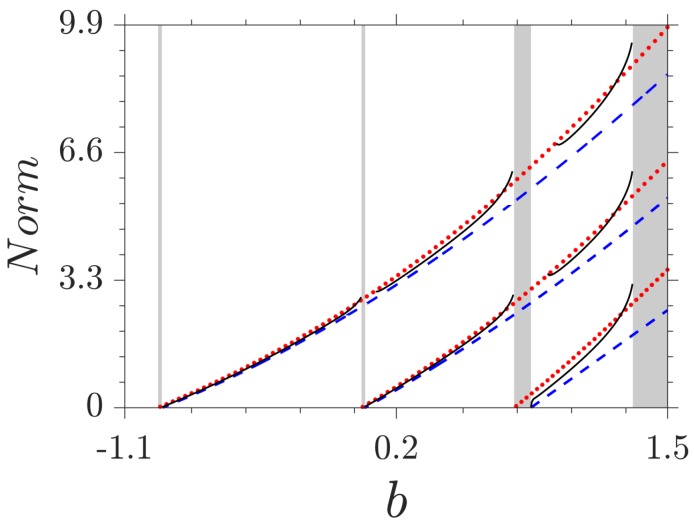
Norms of NLBWs and FGSs versus *b*. Dotted (red) and dashed (blue) lines represent NLBWs originating from the lower and upper edges of linear bands, respectively. Solid (black) lines stands for the norm of gap solitons.

The norms of NLBWs originating from the centers and edges of the BZ for the first three bands are illustrated in Figure 6. With the growth of *b*, all branches of norms increase monotonously and the difference between the norms of NLBWs bifurcating from the centers and edges of the BZ becomes apparent. The existence domains of NLBWs is $b\in [b_{\mathrm{cr}},\infty )$ with $b_{\mathrm{cr}}$ being the lower and upper band edges of the corresponding linear fractional system. They do not feel the bands and gaps of the linear system, in sharp contrast to the localized solitons, which are restricted completely in the bandgaps of the linear system.

Considering the fact that the profiles of FGSs used as unit cells in the *n*th gap have n−1 nodes (Figure 4 and Figure 5), there should exist *n* families of FGSs and 2n families of NLBWs in the *n*th gap. The first family (lowest branches) of FGSs bifurcate from the LBWs at the edges of the BZ and are thresholdless. However, the upper branches of FGSs have lower cutoffs of norms, below which no FGSs can be found. According to the Vakhitov-Kolokolov criterion [27], the inflexions on the norm curves indicate that such nonlinear modes are expected to be linearly unstable at dN/db<0. The norms between NLBWs and FGWs are very close, especially in the first two gaps.

In the system with a fixed lattice and phase mask (fractional-order diffraction effect), when broad (modulated) beams with patterns resembling those shown in Figure 4 and Figure 5 covering many lattice periodicities are input, the optical field far from the boundaries can be viewed as NLBWs experimentally. Changing the distribution, amplitude and width of the input beams will lead to the excitation of different families of NLBWs, as well as truncated-Bloch-wave solitons and FGSs.

**Figure 7:**
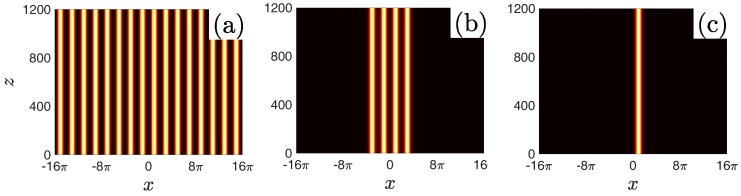
Numerical propagation simulation of NLBW (**a**), truncated-Bloch-wave soliton (**b**) and FGS (**c**) shown in Figure 4b.

To illustrate the propagation behaviour of the delocalized and localized nonlinear modes, we numerically perform the propagation simulation of NLBWs, truncated-Bloch-wave solitons and FGSs by the pseudospectral method put forward by Yang [26]. Representative examples are shown in Figure 7. Stable nonlinear modes can propagate over an arbitrary distance without any deformations.

Finally, we note that the composition relation between NLBWs and FGSs holds in higher gaps. We stress that it can be applied to the system with other forms of periodic potentials and any value of the Lévy index (1<α≤2). Our results can also be generalized into the two-dimensional and three-dimensional cases, just similar to the truncated-Bloch-wave solitons in [5]. We also mention that the term “nonlinear Bloch bands” in [7] may not be exact, since NLBWs exist continuously provided $b\geq b_{\mathrm{cr}}$ and there are no nonlinear gaps separating them (Figure 6).

## 5. Conclusions

To summarize, we studied for the first time the properties of nonlinear Bloch waves supported by nonlinear fractional systems with a periodic potential. Extended periodic nonlinear states in a defocusing nonlinearity originate from the linear Bloch modes of the corresponding linear systems. They can be viewed as a chain of fundamental gap solitons with the same or alternatively opposite signs. The fundamental gap solitons reside mainly in one lattice channel and have n−1 nodes and *n* peaks in the *n*th gap. Additionally, we demonstrate that truncated-Bloch-wave solitons bridge the fundamental gap solitons and nonlinear Bloch waves very well, no matter what value of the Lévy index is.

## Abbreviations

The following abbreviations are used in this manuscript:

NLBWs	nonlinear Bloch waves
LBWs	linear Bloch waves
BZ	Brillouin zone
BECs	Bose-Einstein condensates
NLSE	nonlinear Shrödinger equation
GPE	Gross-Pitaevskii equation
FSE	fractional Schrödinger equation
FGSs	fundamental gap solitons
NLFSE	nonlinear fractional Schrödinger equation
